# The clinical significance, immune infiltration, and tumor mutational burden of angiogenesis-associated lncRNAs in kidney renal clear cell carcinoma

**DOI:** 10.3389/fimmu.2022.934387

**Published:** 2022-07-26

**Authors:** Wei Zhang, Zhiming Liu, Jinpeng Wang, Bo Geng, Wenbin Hou, Enyang Zhao, Xuedong Li

**Affiliations:** Department of Urology, The Second Affiliated Hospital of Harbin Medical University, Harbin, China

**Keywords:** angiogenesis-associated genes, lncRNA, KIRC, independent prognostic predictor, treatment biomarkers

## Abstract

**Background:**

Poor prognosis of kidney renal clear cell carcinoma (KIRC) is often related to angiogenesis. The lncRNAs that regulate angiogenesis could also affect the prognosis of KIRC. It is meaningful for us to use lncRNAs related to angiogenesis to construct a generic, individualized prognostic signature for patients with KIRC.

**Methods:**

We identified eight angiogenesis-associated genes (AAGs) by differential expression analysis and univariate Cox regression from The Cancer Genome Atlas dataset, including 537 KIRC samples and 72 normal samples. In total, 23 prognostic lncRNAs were screened out after Pearson correlation analysis and univariate Cox regression analysis. Then, we performed least absolute shrinkage and selection operator (LASSO) regression and multivariate Cox regression to establish a four-AAG-related lncRNA prognostic signature.

**Results:**

The risk score was calculated for each KIRC patients by using a four-AAG-related lncRNA prognostic signature. We divided the KIRC patients into high- and low-risk groups by the median of the risk score. It was confirmed that the AAG-related lncRNA prognostic signature has good prognostic value for KIRC patients by time-dependent receiver operating characteristic and Kaplan–Meier survival analysis. We identified 3,399 differentially expressed genes between the high- and low-risk groups and performed their functional enrichment analyses. The AAG-related lncRNA prognostic signature was an independent prognostic predictor for KIRC patients and was used to perform a combined nomogram. We reevaluated them in terms of survival, clinic characteristics, tumor-infiltrating immune cells and tumor mutation burden.

**Conclusion:**

Our research indicates that the AAG-related lncRNA prognostic signature is a promising and potential independent prognostic indicator for KIRC patients. Then, it could offer new insights into the prognosis assessment and potential treatment strategies of KIRC patients.

## Introduction

Renal cell carcinoma (RCC) is the third most common urologic cancer, with an annual global incidence of more than 400,000 and a mortality rate of more than 170,000 (1). RCC is classified into different histopathological subtypes based on a specific molecular pattern. KIRC is the most common histopathological subtype, accounting for 75% of all RCC cases (2). KIRC could not be diagnosed early, resulting in the poor efficacy of conventional treatment and low survival rate (3). Molecularly targeted therapies, including anti-vascular endothelial growth factors, have made therapeutic advances, but improving patients’ overall survival (OS) and progression-free survival (PFS) remains a major challenge (4, 5). The development and metastasis of malignant tumors require the establishment of an adequate blood supply, that is, tumor angiogenesis (6). During angiogenesis, pro-angiogenic growth factors are highly expressed in tumor cells (7). Therefore, it is necessary to identify some new effective angiogenic gene signatures for KIRC.

lncRNAs have been found to play key roles in cell growth, cell cycle, apoptosis, cell differentiation, cell invasion, and metastasis (8–11). Abnormally expressed lncRNAs are closely related to various diseases, such as tumor occurrence and development (12–15). Recently, some independent studies have shown that dysregulation of lncRNAs affects tumor angiogenesis (16, 17). The lncRNA RPL34-AS1 regulates the angiogenic gene VEGFA to promote proliferation and angiogenesis in glioma (18). The lncRNA MALAT1 affects the miR-101-3p/STC1 axis to promote the development of colon cancer (19). Currently, few studies have explored the underlying mechanisms of angiogenic lncRNAs for the initiation, progression, and treatment of KIRC. Therefore, exploring unclear correlations between angiogenesis-related genes and lncRNAs may help identify biomarkers as useful therapeutic targets for KIRC.

In this research, we constructed a new AAG-related lncRNA prognostic signature from the TCGA dataset for the KIRC. We used the ROC analysis to confirm that the signature has a high prognostic value. The prognostic signature of AAG-related lncRNAs was well validated in different clinical features and stratified analyses. The AAG-related lncRNA prognostic signature was closely related with tumor-infiltrating immune cells (TICs) and tumor mutation burden (TMB). The AAG-related lncRNA prognostic signature will provide a theoretical basis for better realization of precision targeted therapy in clinical practice with KIRC patients.

## Materials and methods

### Data acquisition

The transcriptome RNA-seq data of 609 KIRC cases (KIRC samples, 537 cases; normal samples, 72 cases) and related clinical information were obtained from The Cancer Genome Atlas (TCGA) dataset (https://portal.gdc.cancer.gov/). To ensure valid analyses, we retained samples with survival time ≥30 days. In total, 36 AAGs were obtained from the MSigDB Team (Hallmark Gene set) (20) (**Supplementary Table S1**).

### Eight AAGs in KIRC acquisition

The 14 AAGs which were differentially and highly expressed in KIRC that were in tumor samples relative to normal samples were determined (*p* < 0.05, logFC > 1) (**Supplementary Table S2**). According to the 14 AAGs, the univariate Cox regression analysis by R package “survival” (21) (*p* < 0.05) showed the eight AAGs which were significantly correlated with KIRC prognosis.

### Four AAG-related lncRNAs of prognostic signature obtainment

To identify AAG-related lncRNAs, we firstly acquired all lncRNA expression data according to the GENCODE project (http://www.gencodegenes.org) in the TCGA dataset. We used the Pearson correlation analysis to identify the AAG-related lncRNAs between AAGs and all lncRNAs based on the correlation coefficient and *p*-values (|Corpearson| > 0.5 and *p* < 0.01). Then, we identified 23 AAG-related lncRNAs by univariate Cox regression (*p* < 0.001). We used the R package “glmnet” (22) with the minimum 10-fold cross-validation (23) to perform the LASSO regression. Lastly, we used multivariate Cox regression to obtain an AAG-related lncRNA prognostic signature for the KIRC patients involving four AAG-related lncRNAs (*p* < 0.05).

### RNA extraction and quantitative real-time PCR

We extracted total RNA from 786O and 293T cells by the TRIzol reagent (Life Technologies, Thermo Fisher Scientific, USA). We used All-in-one First Strand cDNA Synthesis Kit (Seven Bio Inc., Beijing, China) to synthesize the complementary DNA and used 2× SYBR Green qPCR MasterMix (Seven Bio Inc., Beijing, China) to perform quantitative real-time PCR (qRT-PCR) following the standard protocol (24). The forward primer for AC093278.2 was 5′-GCAAGCTTTGTGGGAAGGAA-3′, and the reverse primer for AC093278.2 was 5′-TGGGCAATAGAGGCACTTGA-3′. The forward primer for NNT-AS1 was 5′-CTGGAATCCCTGCTACTCAGGA-3′, and the reverse primer for NNT-AS1 was 5′-GCCATGTGATATGCCTGCTC-3′. The forward primer for CYTOR was 5′-TGGGAATGGAGGGAAATAAA-3′, and the reverse primer for CYTOR was 5′-CCAGGAACTGTGCTGTGAAG-3′. The forward primer for NUP50-DT was 5′-CTGGAAGTTAGAGCTGAGGAAGTT-3′, and the reverse primer for NUP-50DT was 5′-GGGAAATAATAAGGGCTCAGGAAGG-3′. The forward primer for GAPDH was 5′-CATGTTCGTCATGGGTGTGAA-3′, and the reverse primer for GAPDH was 5′-GGCATGGACTGTGGTCATGAG-3′. GAPDH served as the control. The relative expression was calculated by the 2^−△△Ct^ method.

### Non-negative matrix factorization clustering

KIRC samples were clustered by applying non-negative matrix factorization (NMF) clustering algorithm *via* the R package “NMF” to explore potential subgroups (25). We set the number of clusters *k* from 2 to 9. Lastly, due to the cophenetic correlation coefficients, the best *k* = 2 was chosen.

### Screening of prognostic-related lncRNAs and verification of a prognostic model

The risk score is the lncRNA expression for each prognosis multiplied by the lncRNA coefficient for each prognosis: risk score = AC093278.2 × (-0.351782815872485) + NNT-AS1× (-0.336893752787579) + CYTOR × (0.256677130521836) + NUP50-DT × (0.584700743765635). KIRC patients were divided into high- and low-risk groups according to the median cutoff of the risk score from the R packages “survival”, “pheatmap” (26), and “ggupbr” (27). We used the Kaplan–Meier survival curve analysis with log-rank test and time-dependent ROC analysis to analyze OS and to evaluate the accuracy of model predictions. Principal component analysis (PCA) has demonstrated the expression of KIRC samples. Chi-square test was used to analyze the relationship between clinical characteristics and prognostic models. We performed univariate and multivariate Cox regression analyses between the risk score and clinical characteristics to confirm that the prognostic model was an independent predictor of clinical prognosis. In addition, a nomogram was established, using the independent prognostic predictors, by the R package “rms” (26).

### GO and KEGG enrichment analysis

Gene Ontology (GO) and Kyoto Encyclopedia of Genes and Genomes (KEGG) enrichment analyses were performed by the R packages “clusterProfiler” (28), “enrichplot” (28), and “ggplot2” (29). Both *p*- and *q*-values <0.05 were considered significantly enriched.

### Immune microenvironment analysis

The CIBERSORT algorithm was used to acquire the TICs content of the tumor gene expression dataset. Then, we tested the difference between risk groups defined by the prognostic signature using a two-sample *t*-test. Moreover, the R package “ggpubr” (27) was used to exhibit the relationship between immune checkpoints and different risk groups.

### Mutation analysis

We achieved the mutation data of KIRC patients from the TCGA dataset (https://portal.gdc.cancer.gov/). Then, we used the R package “maftools” (30) to analyze and summarize the data containing somatic variants. The TMB score was measured by the formula: (total mutation/total covered bases) × 10^6^.

### Statistical analysis

The prognostic differences between the groups were examined using the Kaplan–Meier survival curves analysis, and the *p*-value was checked in the log-rank test. Univariate and multivariate Cox regression analyses were conducted to illustrate the relationship between the risk score and clinical characteristics. The ROC curves evaluated the value of the risk score for prognosis prediction, and we used the area under the ROC curve as an indicator of prognostic accuracy. Pearson’s correlation test was used for correlation analysis. We used R software (version 4.0.3) for statistical analysis and used Strawberry Perl programming language (version 5.30.1) for data processing (***p < 0.001, **p < 0.01, and *p < 0.05).

## Results

### Identification of eight AAGs in KIRC patients

**Figure 1:**
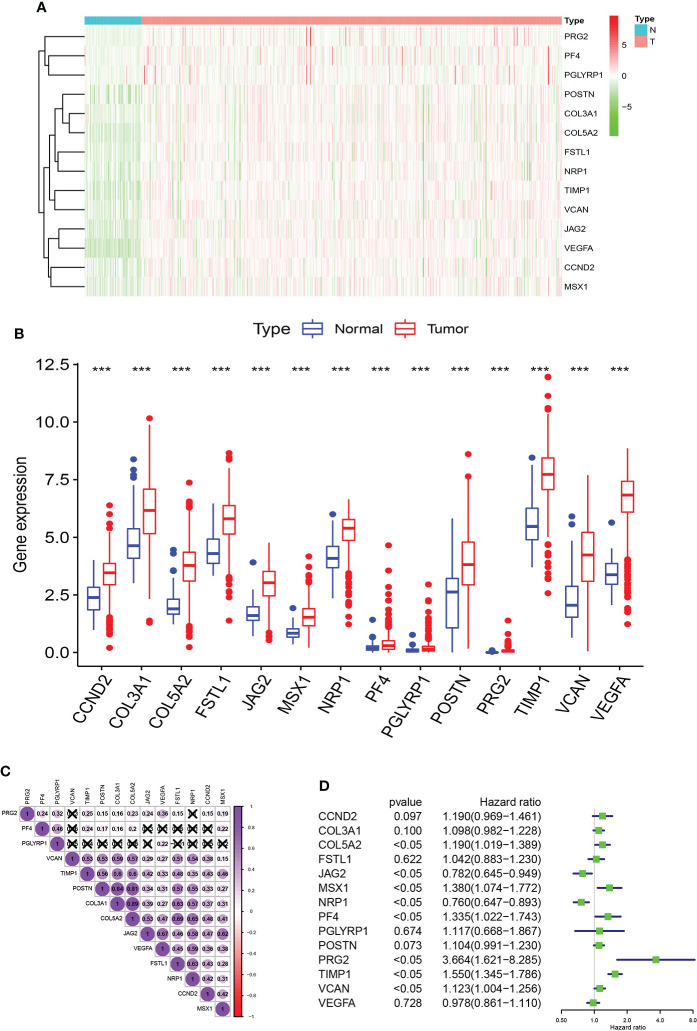
AAGs screening. Heat map **(A)** and box plot **(B)** showing the expression distributions of differentially expressed genes between KIRC and normal samples of the AAGs. **(C)** Correlation analysis of the 14 AAGs. **(D)** Univariate Cox regression analysis of 14 AAGs. The meaning of the symbol *** is p<0.001.

Firstly, we acquired the transcriptome profiling data through the KIRC projects of the TCGA dataset, including 537 KIRC samples and 72 normal samples. Next, we used Ensemble’s gene transfer format file to annotate the data and then extracted the expression matrix of 36 AAGs from TCGA. In total, 14 different AAGs which were differentially and highly expressed in KIRC were identified due to their expression levels in the KIRC samples and the normal samples (**Figures 1A**, **B**). The 14 different AAGs included CCND2, COL3A1, COL5A2, FSTL1, JAG2, MSX1, NRP1, PF4, PGLYRP1, POSTEN, PRG2, TIMP1, VCAN, and VEGFA (*p* < 0.05, logFC>1). The correlations among these 14 AAGs are shown in **Figure 1C**. Lastly, we used univariate Cox regression analysis to evaluate the prognostic effect of 14 AAGs. The forest plot showed that JAG2 and NRP1 were protective factors with hazard ratio (HR) <1 (*p*<0.05), while COL5A2, MSX1, PF4, PRG2, TIMP1, and VCAN were risk factors with HR > 1 (*p* < 0.05) in KIRC patients (**Figure 1D**). The abovementioned results showed that the eight AAGs played an essential biological role in the development of KIRC patients.

### Exploration of the prognostic AAG-related lncRNAs in KIRC

**Figure 2:**
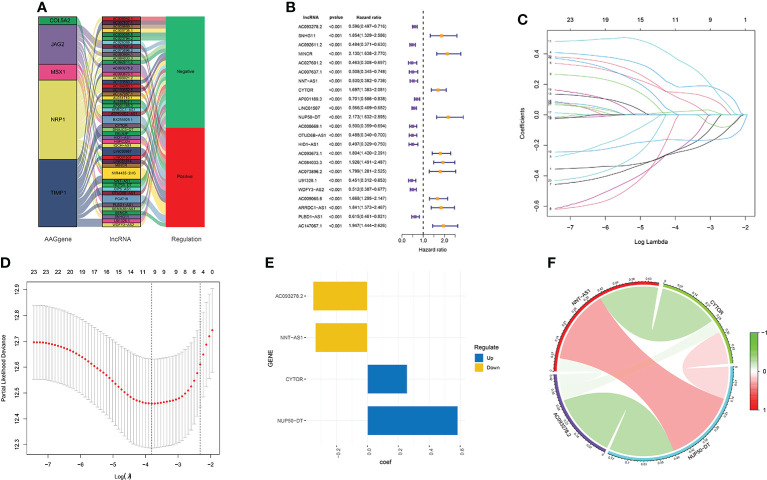
Screening of the four AAG-related prognostic lncRNAs. **(A)** Association between AAGs and targeted lncRNAs. **(B)** Univariate Cox regression analysis of 23 AAG-related lncRNAs (*p* < 0.001). **(C)** LASSO coefficient profiles of 23 AAG-related lncRNAs. **(D)** LASSO regression with 10-fold cross-validation obtained nine AAG-related lncRNAs using a minimum lambda value. **(E)** Multivariate Cox regression of the four prognostic AAG-related lncRNAs. **(F)** Correlation analysis of the four AAG-related lncRNAs.

According to the eight AAGs, we used the Pearson coefficient and *p*-value (|Corpearson| > 0.5 and *p* < 0.01) to acquire the AAGs significantly related to lncRNAs. The Sankey diagram showed the relationship between AAGs and 47 targeted lncRNAs (**Figure 2A**). The 47 AAG-related lncRNAs were included in the univariate Cox regression analysis, and 23 prognostic lncRNAs demonstrated their prognostic roles (*p* < 0.001) (**Figure 2B**). To construct the AAG-related lncRNA prognostic signature for forecasting the OS of KIRC patients, we performed a LASSO Cox regression analysis due to the 23 AAG-related prognostic lncRNAs, and it generated the AAG-related lncRNA prognostic signature which contains nine AAG-related lncRNAs and the coefficient of each (**Figures 2C, D**). Lastly, we used the multivariate Cox regression to screen the AAG-related lncRNAs with the greatest prognostic value. The four AAG-related lncRNAs include AC093278.2, NNT-AS1, CYTOR, and NUP50-DT (*p*<0.05) that were identified to construct the prognostic model for KIRC patients (**Figure 2E**). The correlations among these four AAG-related lncRNAs are shown in **Figure 2F**.

### Exploration of the expression of the four AAG-related lncRNAs in KIRC

**Figure 3:**
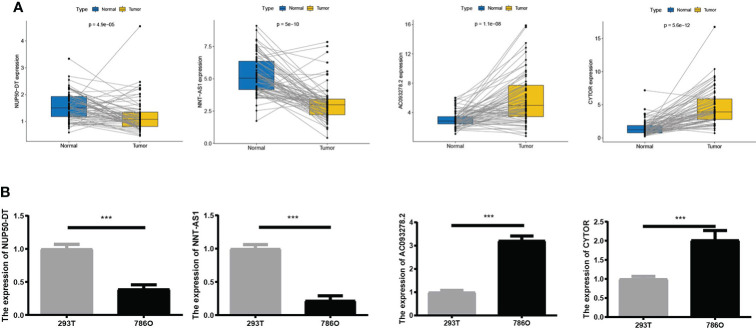
**(A)** Expression of four lncRNAs in KIRC and normal samples from The Cancer Genome Atlas. **(B)** A qRT-PCR analysis was conducted to detect the expression levels of four lncRNAs among 786O and 293T cells. The meaning of the symbol *** is p<0.001.

We compared the expression levels of four AAG-related lncRNAs in KIRC and normal samples through the TCGA dataset and found that AC093278.2 and CYTOR showed higher expression levels in the KIRC samples compared to the normal samples, while NNT-AS1 and NUP50-DT showed lower expression levels in the KIRC samples compared to the normal samples (**Figure 3A**). The expression levels of four AAG-related lncRNAs in 786O and 293T cells were evaluated by qRT-PCR analysis and found to be consistent with the TCGA results (**Figure 3B**).

### Two molecular subgroups of KIRC divided from NMF clustering

**Figure 4:**
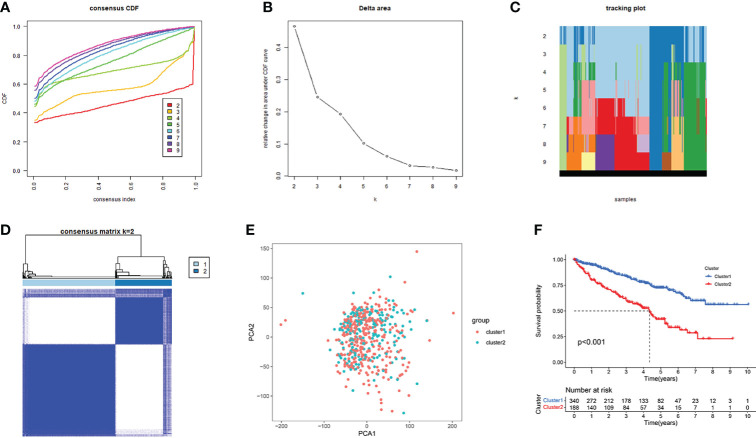
Consensus clusters by 23 AAG-related lncRNAs. **(A)** Consensus clustering cumulative distribution function (CDF) for *k* = 2 to 9. **(B)** Relative change in area under the CDF curve for *k* = 2 to 9. **(C)** Tracking plot for *k* = 2 to 9. **(D)** Consensus clustering matrix for *k* = 2. **(E)** Principal component analysis of the gene expression profiles. **(F)** Kaplan–Meier curve showing a different prognosis between the two clusters.

We selected AAG-related lncRNAs with significant survival differences from the results of the univariate Cox regression analysis to explore the potential molecular subgroups of KIRC. A total of 528 KIRC patients with 23 lncRNAs were used in the NMF consensus clustering analysis. Moreover, *k* = 2 was determined as the optimal *k* value by cophenetic correlation coefficients (**Figures 4A**–**C**). The KIRC samples were divided into cluster 1 (*n* = 340) and cluster 2 (*n* = 188) (**Figure 4D**). We found significant differences in the gene expression profiles between cluster 1 and cluster 2 by PCA (**Figure 4E**). Moreover, the Kaplan–Meier survival curves showed that cluster 1 had a better OS than cluster 2 in KIRC patients (*p* < 0.001) (**Figure 4F**). The abovementioned results not only showed that the KIRC patients could be divided into two subgroups but also identified their differences in OS. Our results showed that subgroups defined by AAG-related lncRNA expression had a close relationship with the heterogeneity of KIRC patients.

### Construction and validation of the AAG-related lncRNA prognostic model in KIRC

**Figure 5:**
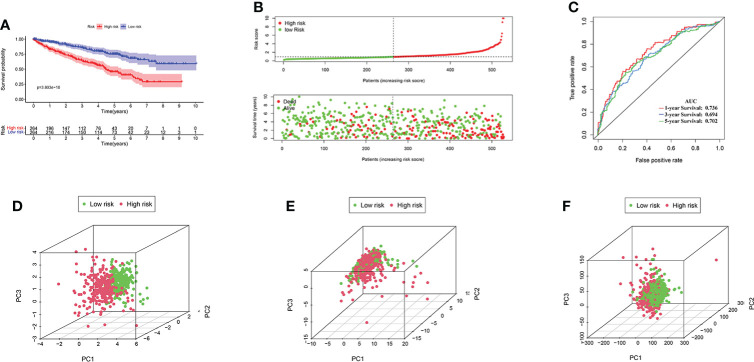
**(A)** KIRC patients in the high-risk group had a worse overall survival than the low-risk group by Kaplan–Meier curves. **(B)** The distribution of risk score and survival times of KIRC patients. **(C)** Receiver operating characteristic analysis of the angiogenesis-associated gene (AAG)-related lncRNA prognostic signature for predicting the 1/3/5-year survival. **(D)** Principal component analysis among high- and low-risk groups based on the four prognostic AAG-related lncRNA sets. **(E)** PCA among high- and low-risk groups based on all the AAG-related lncRNA sets. **(F)** PCA among high- and low-risk groups based on the whole gene sets.

Excluding the KIRC samples with incomplete clinical information, the coefficients of four AAG-related prognostic lncRNAs were used to calculate the risk score of each patient. According to the determined cutoff point, there were 264 cases in the high-risk group and the low-risk group, respectively. The Kaplan–Meier analysis showed that low-risk KIRC patients had a higher OS than high-risk KIRC patients (*p* < 0.001; **Figure 5A**). The risk scores and survival of each case showed that the clinical outcomes of patients in the low-risk group were better than those in the high-risk group (**Figure 5B**). Moreover, the four AAG-related prognostic lncRNAs showed great AUC values in a time-dependent ROC analysis (**Figure 5C**), which meant that the AAG-related lncRNA prognostic model had better prediction ability of the 1-, 3-, and 5-year OS. The Kaplan–Meier survival curves showed that not only the high expression of AC093278.2 and NNT-AS1 but also the low expression of CYTOR and NUP50-DT were associated with better OS in the TCGA dataset (**Supplementary Figure S1**). Different distribution patterns between the high- and low-risk groups were detected by PCA. The PCA results based on the prognostic model genome showed a significant difference between the high-risk and the low-risk groups (**Figure 5D**), while we did not detect a significant separation on the basis of the AAG-related lncRNAs and the genome-wide expression profiles (**Figures 5E, F**). To sum up, the four AAG-related prognostic lncRNAs performed well in the prediction of OS in KIRC patients.

### Clinical evaluation by the AAG-related lncRNA prognostic model

The heat map shows the relationship between the risk score of KIRC and clinical characteristics (**Supplementary Figure S2A**). Then, consequent scatter diagrams obtained by the Wilcoxon signed-rank test showed that tumor grade, clinical stage, T stage, N stage, and M stage (**Supplementary Figures S2B–F**) were positively related to the risk score, while age and gender (**Supplementary Figures S2G, H**) were not significantly related to the risk score. The abovementioned results confirmed that KIRC had a higher risk score and a higher degree of malignancy, regardless of age and gender.

### The AAG-related lncRNA prognostic signature was an independent prognostic predictor for KIRC patients

**Figure 6:**
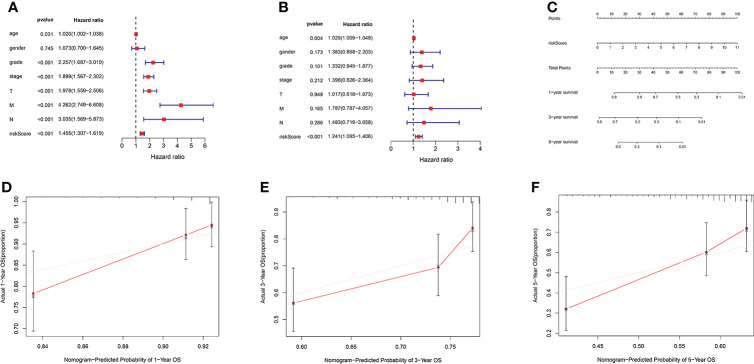
**(A)** Forest plot for univariate Cox regression analysis showing that grade, stage, T stage, M stage, N stage, and risk score were prognostic risk-related variables (*p* < 0.001). **(B)** Forest plot for multivariate Cox regression analysis showing that only the risk score was the independent prognostic factor (*p* < 0.001). **(C)** Nomogram integrating the risk score of four AAG-related lncRNAs. **(D–F)** Calibration curve analysis of the nomogram for predicting the 1-, 3-, and 5-year overall survival in The Cancer Genome Atlas dataset.

We used univariate and multivariate Cox regression analyses to assess independent prognostic predictors in KIRC patients. The univariate Cox regression analysis showed that the AAG-related lncRNA prognostic signature had a close relationship with OS (HR: 1.324, 95% CI: 1.211–1.449, *p* < 0.001) (**Figure 6A**), and the multivariate Cox regression analysis also further showed that the AAG-related lncRNA prognostic signature was remarkably associated with OS (HR: 1.160, 95% CI: 1.041–1.293, *p* < 0.001) (**Figure 6B**). We established a nomogram using the AAG-related lncRNA prognostic signature screened by univariate and multivariate Cox regression analyses (**Figure 6C**). The calibration plots showed high concordance in predicting the 1-, 3-, and 5-year OS in KIRC patients (**Figures 6D**–**F**). These results showed that, as the only independent prognostic predictor, the AAG-related lncRNA prognostic signature may be useful for clinical prognostic evaluation.

### Pathway and process enrichment analysis

**Figure 7:**
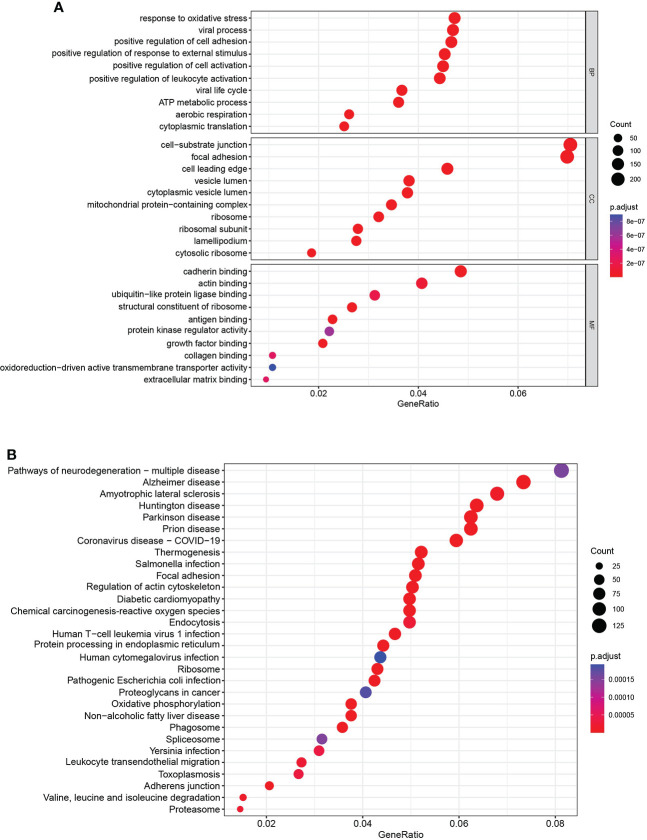
Functional enrichment analysis. **(A)** Gene Ontology analysis of DEGs revealed the enriched biological processes, cell components, and molecular functions. **(B)** Kyoto Encyclopedia of Genes and Genomes pathway analysis of DEGs revealed the enriched signaling pathways.

To explore the potential biological pathway and process involved in the molecular heterogeneity between the high- and low-risk groups, we identified 3,399 differentially expressed genes (DEGs) [|log2 (fold change)| > 2 and *p* < 0.05] between the high- and low-risk groups in KIRC patients. GO enrichment analysis and KEGG pathway analysis of DEGs were adopted. We found that the top five GO terms for biological processes were response to oxidative stress, viral process, positive regulation of cell adhesion, positive regulation of response to external stimulus, and positive regulation of cell activation. The top five GO terms for cellular components were cell−substrate junction, focal adhesion, cell leading edge, vesicle lumen, and cytoplasmic vesicle lumen. The top five GO terms for molecular functions were cadherin binding, actin binding, ubiquitin-like protein ligase binding, structural constituent of ribosome, and antigen binding (**Figure 7A**). According to the KEGG analysis, the top five pathways included pathways of neurodegeneration-multiple disease, Alzheimer disease, amyotrophic lateral sclerosis, Huntington’s disease, and Parkinson’s disease (**Figure 7B**). These abovementioned results may give us some insights into the cellular biological effects related to the AAG-related lncRNA prognostic signature.

### The relationship between immune microenvironment and risk score

**Figure 8:**
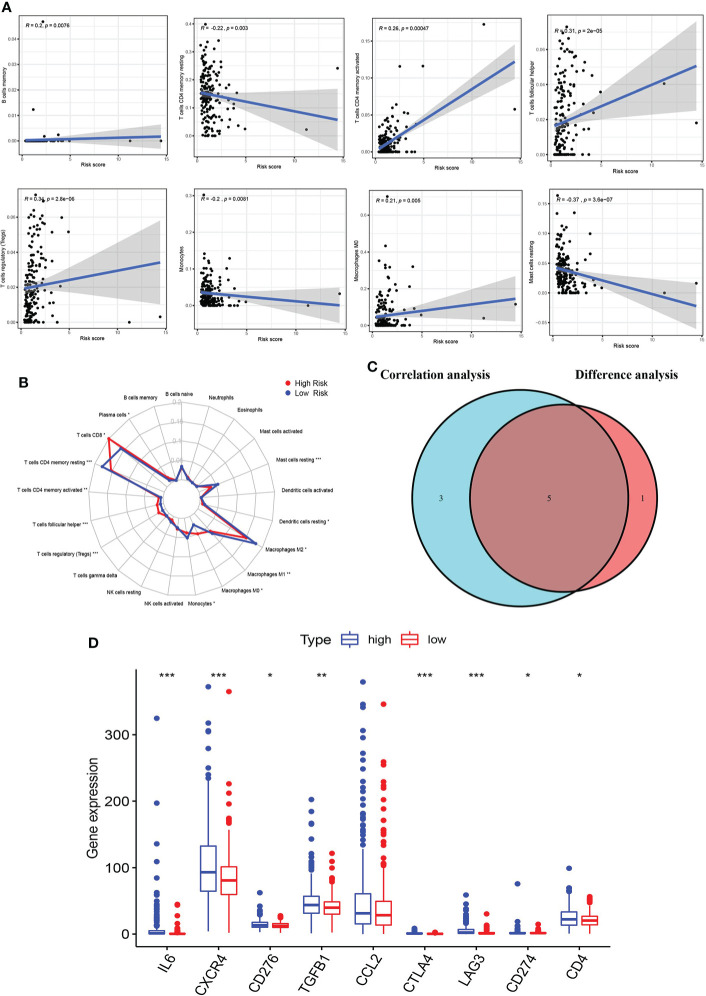
Correlation of immune microenvironment with risk score. **(A)** Scatter plot showing eight significantly correlated TICs (p < 0.01). The blue line in each plot was a fitted linear model indicating the proportion of tropism of the immune cell along with risk score, and Pearson coefficient was used for the correlation test. **(B)** Radar plot showing differences in TICs between the high- and low-risk groups as measured by Wilcoxon rank-sum test. **(C)** Venn diagram showing that the 5 TICs were associated with the risk score jointly determined by the difference and correlation tests shown in the scatter and radar charts, respectively (p < 0.01). **(D)** Box plot showing the correlation between immune checkpoint and risk score. The meaning of the symbol *** is p<0.001.

To explore the relationship between the immune microenvironment and risk score, we analyzed the proportion of tumor-infiltrating immune groups by CIBERSORT algorithm and constructed 21 immune cell profiles in the KIRC samples (**Supplementary Figure S3**). We combined correlation analysis (*p* < 0.01) (**Figure 8A**) and difference analysis (*p* < 0.01) (**Figure 8B**) to obtain a total of five TICs associated with the AAG-related lncRNA prognostic signature risk score (**Figure 8C**). Among them, CD4 memory-activated T cells, follicular helper T cells, and regulatory T cells (Tregs) had a positive correlation with the risk score, while CD4 memory resting T cells and resting mast cells were negatively correlated with risk score. Moreover, compared with the low-risk group, the high-risk group had relatively higher expression levels of immune checkpoints, including IL6, CXCR4, CD276, TGFB1, CTLA4, LAG3, CD274, and CD4 (**Figure 8D**). The abovementioned results suggested that different risk groups had a specific relationship with immune microenvironment. We could formulate treatment methods for KIRC patients with different risk groups through the differences between different risk groups and the immune microenvironment.

### The relationship between risk score and TMB

**Figure 9:**
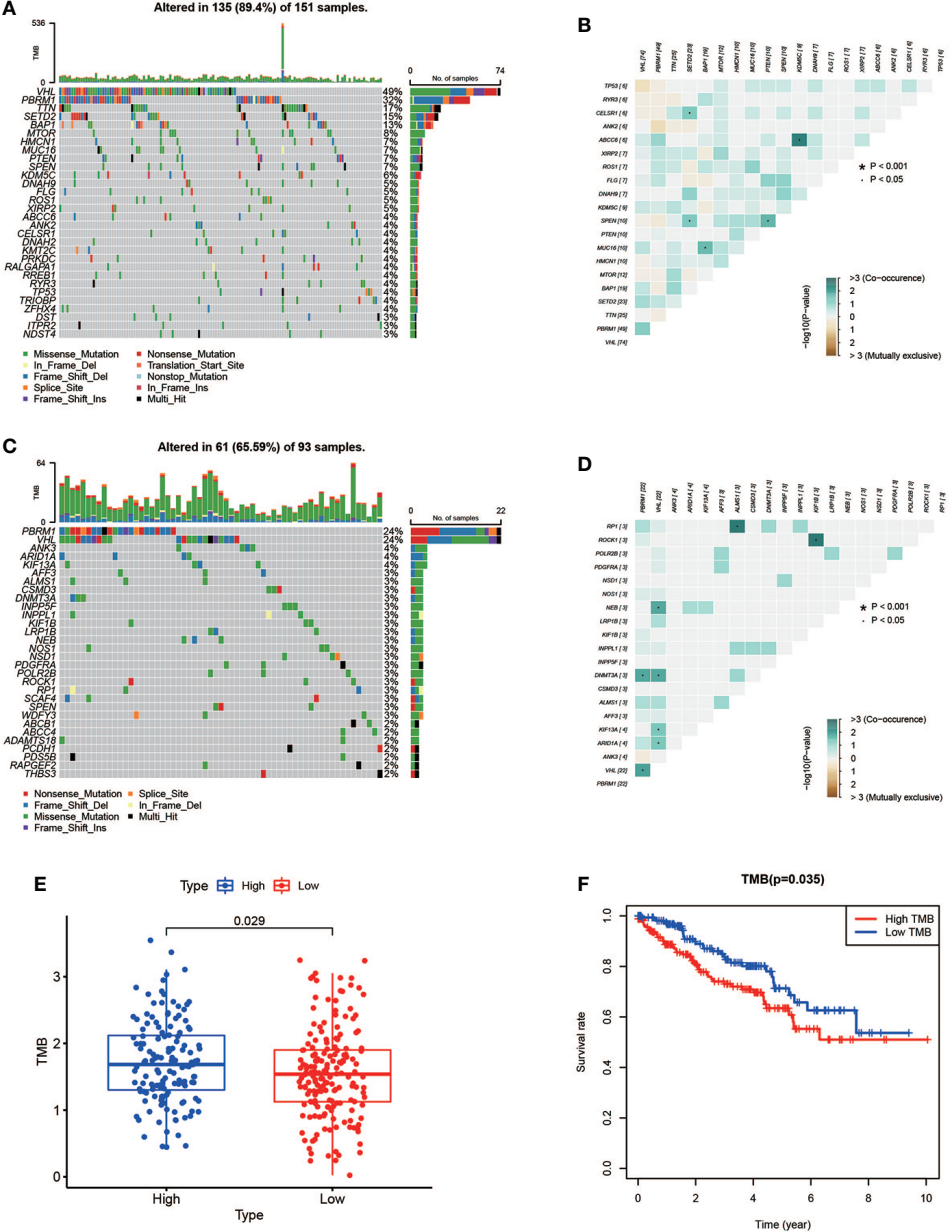
Mutation profile and relationship between tumor mutation burden (TMB) and risk score. **(A, B)** Mutation profile of the high-risk group and interaction among 20 most frequent mutant genes. **(C, D)** Mutation profile of the low-risk group and interaction among 20 most frequent mutant genes. **(E)** Relationship between TMB and risk score. **(F)** Association of TMB and overall survival in KIRC patients.

In the high-risk group, we listed the 20 most frequent mutant genes, including VHL, PBRM1, TTN, SETD2, BAP1, MTOR, HMCN1, MUC16, PTEN, SPEN, KDM5C, DNAH9, FLG, ROS1, XIRP2, ABCC6, ANK2, CELSR1, RYR3, and TP53 and the interaction among them (**Figures 9A**, **B**), while in the low-risk group, PBRM1, VHL, ANK3, ARID1A, KIF13A, AFF3, ALMS1, CSMD3, DNMT3A, INPP5F, INPPL1, KIF1B, LRP1B, NEB, NOS1, NSD1, PDGFRA, POLR2B, POCK1, and RP1 were the 20 most frequent mutant genes, and their interactions are shown in **Figures 9C, D**. A summary of variant classification, variant type, SNV class, and variants per sample in the high- and low-risk groups is shown in **Supplementary Figure S4**. In **Figures 9E, F**, the analysis showed that the high-risk KIRC patients had higher TMB with shorter OS. These data were consistent with previous results obtained with Kaplan–Meier survival curves for the high- and low-risk groups.

## Conclusion

Briefly, we constructed a novel prognostic signature of four AAG-related lncRNAs (AC093278.2, NNT-AS1, CYTOR, and NUP50-DT) for KIRC patients. A series of analyses were performed, and the results indicated that the newly constructed prognostic signature could be a potential predictor for KIRC patients. In summary, our study indicates that the prognostic signature has close relationships with clinical characteristics, TICs, and TMB, which may help to offer a more individualized treatment for KIRC patients.

## Discussion

As one of the most prevalent primary malignant tumors of the urinary system, KIRC has the characteristics of high heterogeneity, poor prognosis, and distant metastasis (4, 31, 32). It is critical to explore the potential predictor for KIRC treatment and prognosis. Angiogenesis is a complex consequence of co-regulation between pro-angiogenic and anti-angiogenic factors, and it is disrupted and dysregulated in cancer (33). Angiogenesis is an important process in cancer pathogenesis and therapy. lncRNAs play an important role in angiogenesis, so new therapeutic targets and drug candidates are needed to inhibit angiogenesis (10).

Recent studies have shown that the lncRNA PAARH promotes hepatocellular carcinoma (HCC) angiogenesis by activating HIF-1α/VEGF signaling (34). JAG1 is involved in angiogenesis, and Linc-OIP5 may regulate JAG1 signaling through YAP1 signaling (35). The lncRNA H22954 inhibits angiogenesis in acute myeloid leukemia by downregulating PDGFA expression (36). The lncRNA MIR31HG accelerates colorectal cancer progression by targeting miR-361-3p to regulate glycolysis and angiogenesis (37). The abovementioned results indicate that lncRNAs regulate angiogenesis, thereby further achieving the effect of tumor treatment, which has attracted more and more attention. So, we constructed a signature based on AAG-related lncRNAs to achieve better personalized treatment and predict the prognosis of KIRC patients.

We constructed the prognostic signature by using four AAG-related lncRNAs (AC093278.2, CYTOR, NNT-AS1, and NUP50-DT) from 537 KIRC patients. Several of these lncRNAs were reported to be associated with cancer progression. lncRNA CYTOR promotes HCC proliferation by targeting the microRNA-125a-5p/LASP1 axis (38). lncRNA NNT-AS1 promotes estrogen-mediated endometrial carcinoma proliferation by regulating miR-30c/NPM1 (39). The lncRNA NNT-AS1 promotes KIRC progression through the miR-137/YBX-1 pathway (40). These results demonstrate that lncRNAs which construct the signature are involved in tumor progression, but there are fewer reports related to angiogenesis. The prognostic signature also provides some theoretical suggestions for these lncRNAs as potential targets and drug candidates for anti-vascular therapy of tumors.

In our study, the ROC analysis result confirmed that the signature had a high prognostic value. In total, 3,399 DEGs were identified between the high- and low-risk groups; then, GO and KEGG analyses were performed. In addition, the signature showed a significant correlation with clinical characteristics, further supporting its prognostic value. We also identified that the AAG-related lncRNAs can potentially be utilized as an independent predictor for the OS in the TCGA dataset. The nomogram composed of the signature showed a high performance in 1, 3, and 5 years, which may help in the analysis of the prognosis of KIRC patients and the choice of treatment. Moreover, the prognostic signature was closely associated with TICs and TMB, suggesting that they could potentially help clinicians design effective individual therapy for KIRC patients.

Although we used a large number of TCGA dataset, our research still had some limitations. We extensively explored the expression and potential prognostic capabilities of the AAG-related lncRNA prognostic signature in KIRC and the roles of these lncRNAs on angiogenesis in KIRC, but the drug-resistant KIRC has not been specifically elucidated. We will also further study the specific mechanism of these lncRNAs affecting angiogenesis in future studies so as to provide a theoretical basis for these lncRNAs to become therapeutic targets as soon as possible.

## Data availability statement

The transcriptome RNA-seq data of KIRC samples and related clinical information were obtained from the TCGA dataset (https://portal.gdc.cancer.gov/). All lncRNAs expression data in the TCGA dataset were according to the GENCODE project (http://www.gencodegenes.org). The mutation data of KIRC patients were from the TCGA dataset (https://portal.gdc.cancer.gov/).

## Author contributions

WZ and ZL were the first to design the study. JW and BG drafted the manuscript. WZ and WH provided technical assistance for the continuation of the experiment. ZL, EZ, and XL participated in the production and improvement of tables and figures. All authors contributed to the article and approved the submitted version.

## Funding

This work was funded by the Scientific Research Project of Heilongjiang Provincial Health and Family Planning Commission (2017-070) and the Second Affiliated Hospital of Harbin Medical University First-Class Discipline First-Class Specialist Construction Project (100123).

## Conflict of interest

The authors declare that the research was conducted in the absence of any commercial or financial relationships that could be construed as a potential conflict of interest.

The reviewer YL declared a shared parent affiliation with the authors to the handling editor at the time of the review.

## Publisher’s note

All claims expressed in this article are solely those of the authors and do not necessarily represent those of their affiliated organizations, or those of the publisher, the editors and the reviewers. Any product that may be evaluated in this article, or claim that may be made by its manufacturer, is not guaranteed or endorsed by the publisher.

## Supplementary material

Supplementary Figure 1 The KIRC patients with different expression levels of the four AAG-related lncRNAs had different overall survival by Kaplan–Meier curves.

Supplementary Figure 2 Prognostic signature of clinical evaluation. A heat map **(A)** along with a scatter diagram showing that grade **(B)**, stage **(C)**, T stage **(D)**, N stage **(E)**, and M stage **(F)** were significantly associated with the risk score, while age **(G)** and gender **(H)** were not significantly related to the risk score.

Supplementary Figure 3 Distribution of tumor-infiltrating immune cells (TICs) in KIRC and correlation analysis. **(A)** Bar plot showing the distribution of TICs in KIRC. **(B)** Correlation analysis of the 21 TICs in KIRC.

Supplementary Figure 4
**(A)** Summary of variant classification, variant type, SNV class, and variants per sample in the high-risk group. **(B)** Summary of variant classification, variant type, SNV class, and variants per sample in the low-risk group.

## References

[1] Bray F Ferlay J Soerjomataram I Siegel RL Torre LA Jemal A. Global cancer statistics 2018: GLOBOCAN estimates of incidence and mortality worldwide for 36 cancers in 185 countries. CA Cancer J Clin (2018) 68(6):394–424. doi: 10.3322/caac.21492 30207593

[2] Lalani AA McGregor BA Albiges L Choueiri TK Motzer R Powles T. Systemic treatment of metastatic clear cell renal cell carcinoma in 2018: Current paradigms, use of immunotherapy, and future directions. Eur Urol (2019) 75(1):100–10. doi: 10.1016/j.eururo.2018.10.010 30327274

[3] Bai S Wu Y Yan Y Shao S Zhang J Liu J. Construct a circRNA/miRNA/mRNA regulatory network to explore potential pathogenesis and therapy options of clear cell renal cell carcinoma. Sci Rep (2020) 10(1):13659. doi: 10.1038/s41598-020-70484-2 32788609 PMC7423896

[4] Sun Z Tao W Guo X Jing C Zhang M Wang Z. Construction of a lactate-related prognostic signature for predicting prognosis, tumor microenvironment, and immune response in kidney renal clear cell carcinoma. Front Immunol (2022) 13:818984. doi: 10.3389/fimmu.2022.818984 35250999 PMC8892380

[5] Liu D Shu G Jin F Qi J Xu X Du Y. ROS-responsive chitosan-SS31 prodrug for AKI therapy via rapid distribution in the kidney and long-term retention in the renal tubule. Sci Adv (2020) 6(41). doi: 10.1126/sciadv.abb7422 PMC7546709 33036968

[6] Zheng W Zhang S Guo H Chen X Huang Z Jiang S. Multi-omics analysis of tumor angiogenesis characteristics and potential epigenetic regulation mechanisms in renal clear cell carcinoma. Cell Commun Signal (2021) 19(1):39. doi: 10.1186/s12964-021-00728-9 33761933 PMC7992844

[7] Han B Zhang H Tian R Liu H Wang Z Wang Z. Exosomal EPHA2 derived from highly metastatic breast cancer cells promotes angiogenesis by activating the AMPK signaling pathway through ephrin A1-EPHA2 forward signaling. Theranostics (2022) 12(9):4127–46. doi: 10.7150/thno.72404 PMC9169374 35673569

[8] Kopp F Mendell JT. Functional classification and experimental dissection of long noncoding RNAs. Cell (2018) 172(3):393–407. doi: 10.1016/j.cell.2018.01.011 29373828 PMC5978744

[9] Shuai Y Ma Z Lu J Feng J. LncRNA SNHG15: A new budding star in human cancers. Cell Prolif (2020) 53(1):e12716. doi: 10.1111/cpr.12716 31774607 PMC6985667

[10] Kumar MM Goyal R. LncRNA as a therapeutic target for angiogenesis. Curr Top Med Chem (2017) 17(15):1750–7. doi: 10.2174/1568026617666161116144744 PMC5421140 27848894

[11] Sun J Sun X Hu S Wang M Ma N Chen J. Long noncoding RNA SNHG1 silencing accelerates hepatocyte-like cell differentiation of bone marrow-derived mesenchymal stem cells to alleviate cirrhosis *via* the microRNA-15a/SMURF1/UVRAG axis. Cell Death Discov (2022) 8(1):77. doi: 10.1038/s41420-022-00850-8 35194023 PMC8863836

[12] Fang Y Fullwood MJ. Roles, functions, and mechanisms of long non-coding RNAs in cancer. Genomics Proteomics Bioinf (2016) 14(1):42–54. doi: 10.1016/j.gpb.2015.09.006 PMC4792843 26883671

[13] Mercer TR Dinger ME Mattick JS. Long non-coding RNAs: insights into functions. Nat Rev Genet (2009) 10(3):155–9. doi: 10.1038/nrg2521 19188922

[14] Zhou Y Wang L Zhang W Ma J Zhang Z Yang M. Identification of epithelial mesenchymal transition-related lncRNAs associated with prognosis and tumor immune microenvironment of hepatocellular carcinoma. Dis Markers 2022 (2022) p:6335155. doi: 10.1155/2022/6335155 PMC8802097 35111268

[15] Shree B Tripathi S Sharma V. Transforming growth factor-Beta-Regulated LncRNA-MUF promotes invasion by modulating the miR-34a Snail1 axis in glioblastoma multiforme. Front Oncol (2021) 11:788755. doi: 10.3389/fonc.2021.788755 35223453 PMC8865078

[16] Xu Y Leng K Yao Y Kang P Liao G Han Y. A circular RNA, cholangiocarcinoma-associated circular RNA 1, contributes to cholangiocarcinoma progression, induces angiogenesis, and disrupts vascular endothelial barriers. Hepatology (2021) 73(4):1419–35. doi: 10.1002/hep.31493 32750152

[17] Niu Y Bao L Chen Y Wang C Luo M Zhang B. HIF2-induced long noncoding RNA RAB11B-AS1 promotes hypoxia-mediated angiogenesis and breast cancer metastasis. Cancer Res (2020) 80(5):964–75. doi: 10.1158/0008-5472.CAN-19-1532 PMC7056556 31900259

[18] Zhang D Jiang H Ye J Gao M Wang X Lu E. A novel lncRNA, RPL34-AS1, promotes proliferation and angiogenesis in glioma by regulating VEGFA. J Cancer (2021) 12(20):6189–97. doi: 10.7150/jca.59337 PMC8425216 34539892

[19] Luan C Li Y Liu Z Zhao C. Long noncoding RNA MALAT1 promotes the development of colon cancer by regulating miR-101-3p/STC1 axis. Onco Targets Ther (2020) 13:3653–65. doi: 10.2147/OTT.S242300 PMC7200234 32431516

[20] Qing X Xu W Liu S Chen Z Ye C Zhang Y. Molecular characteristics, clinical significance, and cancer immune interactions of angiogenesis-associated genes in gastric cancer. Front Immunol (2022) 13:843077. doi: 10.3389/fimmu.2022.843077 35273618 PMC8901990

[21] Diboun I Wernisch L Orengo CA Koltzenburg M. Microarray analysis after RNA amplification can detect pronounced differences in gene expression using limma. BMC Genomics (2006) 7:252. doi: 10.1186/1471-2164-7-252 17029630 PMC1618401

[22] Friedman J Hastie T Tibshirani R. Regularization paths for generalized linear models via coordinate descent. J Stat Softw (2010) 33(1):1–22. doi: 10.18637/jss.v033.i01 20808728 PMC2929880

[23] Tao C Huang K Shi J Hu Q Li K Zhu X. Genomics and prognosis analysis of epithelial-mesenchymal transition in glioma. Front Oncol (2020) 10:183. doi: 10.3389/fonc.2020.00183 32154177 PMC7047417

[24] Xu J Liu Y Liu J Xu T Cheng G Shou Y. The identification of critical m(6)A RNA methylation regulators as malignant prognosis factors in prostate adenocarcinoma. Front Genet (2020) 11:602485. doi: 10.3389/fgene.2020.602485 33343639 PMC7746824

[25] Gaujoux R Seoighe C. A flexible r package for nonnegative matrix factorization. BMC Bioinf (2010) 11:367. doi: 10.1186/1471-2105-11-367 PMC2912887 20598126

[26] Zhang Z Lin E Zhuang H Xie L Feng X Liu J. Construction of a novel gene-based model for prognosis prediction of clear cell renal cell carcinoma. Cancer Cell Int (2020) 20:27. doi: 10.1186/s12935-020-1113-6 32002016 PMC6986036

[27] Whitehead MJ McCanney GA Willison HJ Barnett SC. MyelinJ: an ImageJ macro for high throughput analysis of myelinating cultures. Bioinformatics (2019) 35(21):4528–30. doi: 10.1093/bioinformatics/btz403 PMC6821319 31095292

[28] Yu G Wang LG Han Y He QY. clusterProfiler: an r package for comparing biological themes among gene clusters. OMICS (2012) 16(5):284–7. doi: 10.1089/omi.2011.0118 PMC3339379 22455463

[29] Wang J Yang J. Identification of significant genes with a poor prognosis in skin cutaneous malignant melanoma based on a bioinformatics analysis. Ann Transl Med (2022) 10(8):448. doi: 10.21037/atm-22-1163 35571409 PMC9096380

[30] Mayakonda A Lin DC Assenov Y Plass C Koeffler HP. Maftools: efficient and comprehensive analysis of somatic variants in cancer. Genome Res (2018) 28(11):1747–56. doi: 10.1101/gr.239244.118 PMC6211645 30341162

[31] Poplawski P Boguslawska J Hanusek K Piekielko-Witkowska A. Nucleolar proteins and non-coding RNAs: Roles in renal cancer. Int J Mol Sci (2021) 22(23). doi: 10.3390/ijms222313126 PMC8658237 34884928

[32] Liu H Yang Y. Identification of mast cell-based molecular subtypes and a predictive signature in clear cell renal cell carcinoma. Front Mol Biosci (2021) 8:719982. doi: 10.3389/fmolb.2021.719982 34646862 PMC8503328

[33] Che X Su W Li X Liu N Wang Q Wu G. Angiogenesis pathway in kidney renal clear cell carcinoma and its prognostic value for cancer risk prediction. Front Med (Lausanne) (2021) 8:731214. doi: 10.3389/fmed.2021.731214 34778292 PMC8581140

[34] Wei H Xu Z Chen L Wei Q Huang Z Liu G. Long non-coding RNA PAARH promotes hepatocellular carcinoma progression and angiogenesis via upregulating HOTTIP and activating HIF-1alpha/VEGF signaling. Cell Death Dis (2022) 13(2):102. doi: 10.1038/s41419-022-04505-5 35110549 PMC8810756

[35] Zhu Q Li Y Dong X Yang Y Wang H Guo S. Linc-OIP5 loss regulates migration and invasion in MDA-MB-231 breast cancer cells by inhibiting YAP1/JAG1 signaling. Oncol Lett (2020) 19(1):103–12. doi: 10.3892/ol.2019.11071 PMC6924107 31897120

[36] Li X Rong J Li T Zhou Y Qi X. LncRNA H22954 inhibits angiogenesis in acute myeloid leukemia through a PDGFA-dependent mechanism. Recent Pat Anticancer Drug Discov (2022) 17:427–34. doi: 10.2174/1871526522666220321154949 35319391

[37] Guo T Liu D Peng S Wang M Li Y. A positive feedback loop of lncRNA MIR31HG-miR-361-3p -YY1 accelerates colorectal cancer progression through modulating proliferation, angiogenesis, and glycolysis. Front Oncol (2021) 11:684984. doi: 10.3389/fonc.2021.684984 34485123 PMC8416113

[38] Liu Y Geng X. Long non-coding RNA (lncRNA) CYTOR promotes hepatocellular carcinoma proliferation by targeting the microRNA-125a-5p/LASP1 axis. Bioengineered (2022) 13(2):3666–79. doi: 10.1080/21655979.2021.2024328 PMC8974008 35081873

[39] Shen J Yuan Z Sheng J Feng X Wang H Wang Y. Long non-coding RNA NNT-AS1 positively regulates NPM1 expression to affect the proliferation of estrogen-mediated endometrial carcinoma by interacting. J Cancer (2022) 13(1):112–23. doi: 10.7150/jca.62630 PMC8692688 34976175

[40] Zhou Y Zhang Z Wo M Xu W. The long non-coding RNA NNT-AS1 promotes clear cell renal cell carcinoma progression *via* regulation of the miR-137/ y-box binding protein 1 axis. Bioengineered (2021) 12(1):8994–9005. doi: 10.1080/21655979.2021.1992330 34643163 PMC8806961

