# Supplementary figures and images for: The clinical significance, immune infiltration, and tumor mutational burden of angiogenesis-associated lncRNAs in kidney renal clear cell carcinoma
Source: Front Immunol. 2022 Jul 26;13:934387. doi: 10.3389/fimmu.2022.934387 (PMC9360495; doi:10.3389/fimmu.2022.934387)

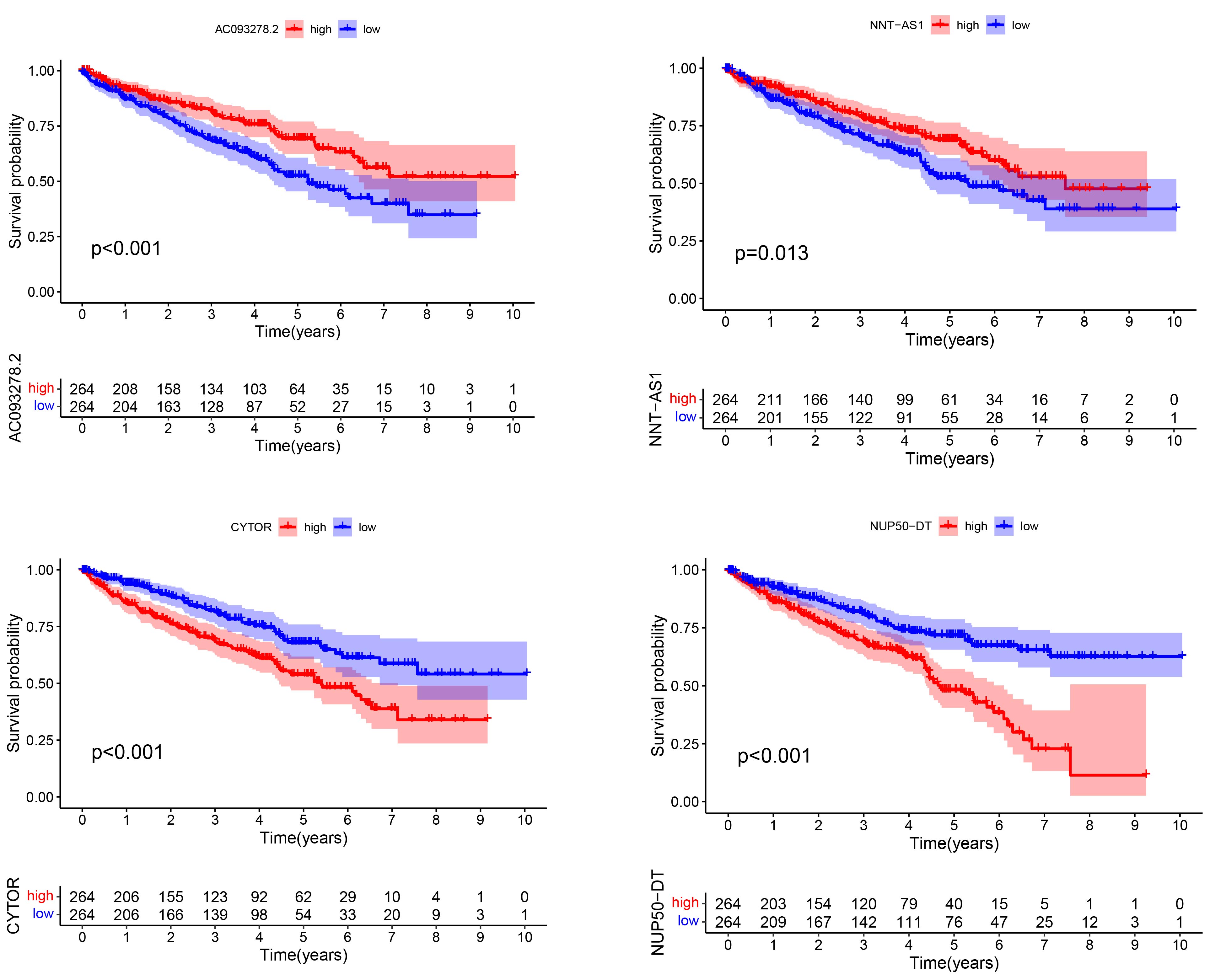

Supplement: Supplementary Figure 1 — The KIRC patients with different expression levels of the four AAG-related lncRNAs had different overall survival by Kaplan–Meier curves. [file Image_1.jpeg]

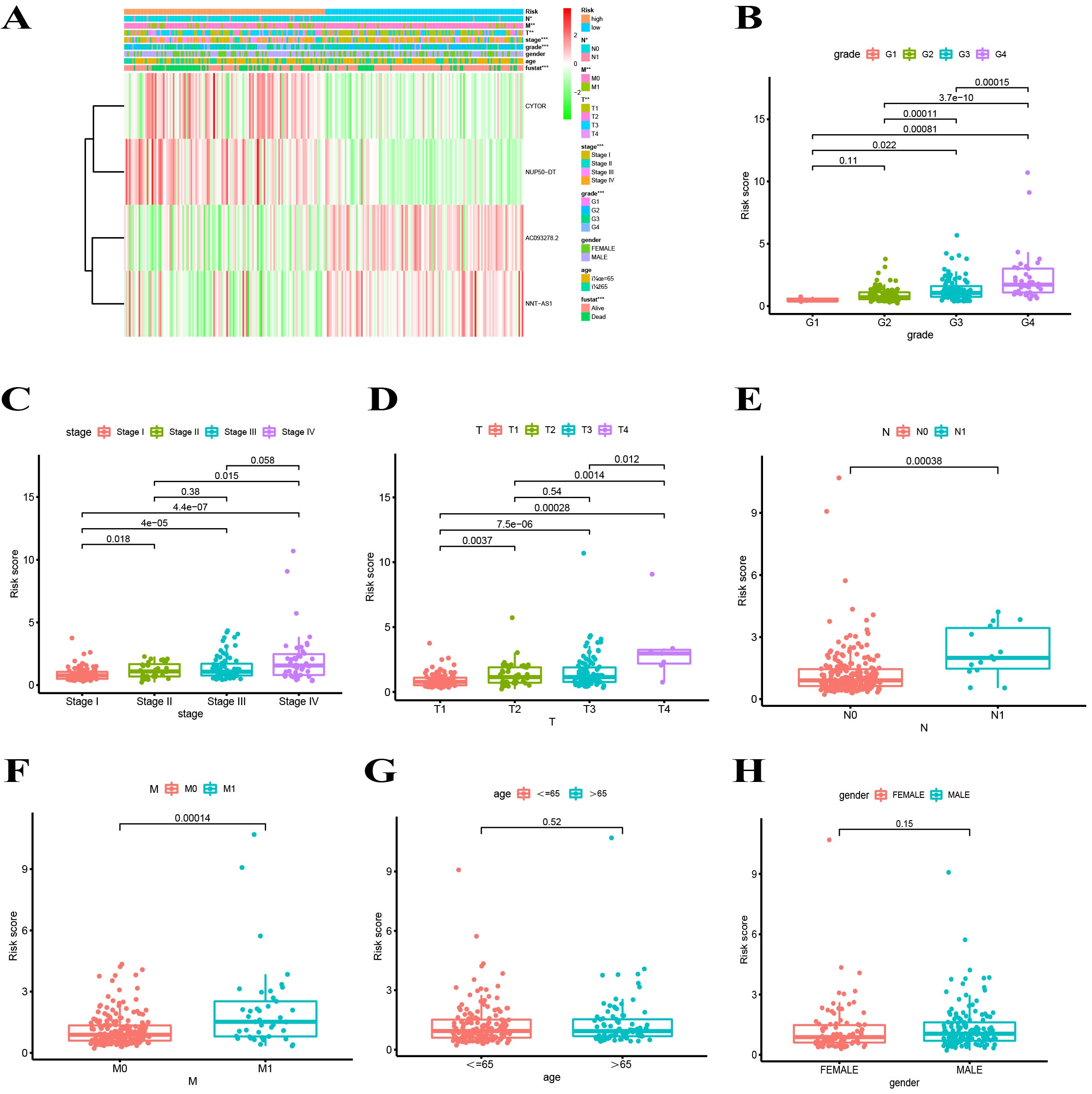

Supplement: Supplementary Figure 2 — Prognostic signature of clinical evaluation. A heat map (A) along with a scatter diagram showing that grade (B), stage (C), T stage (D), N stage (E), and M stage (F) were significantly associated with the risk score, while age (G) and gender (H) were not significantly related to the risk score. [file Image_2.jpeg]

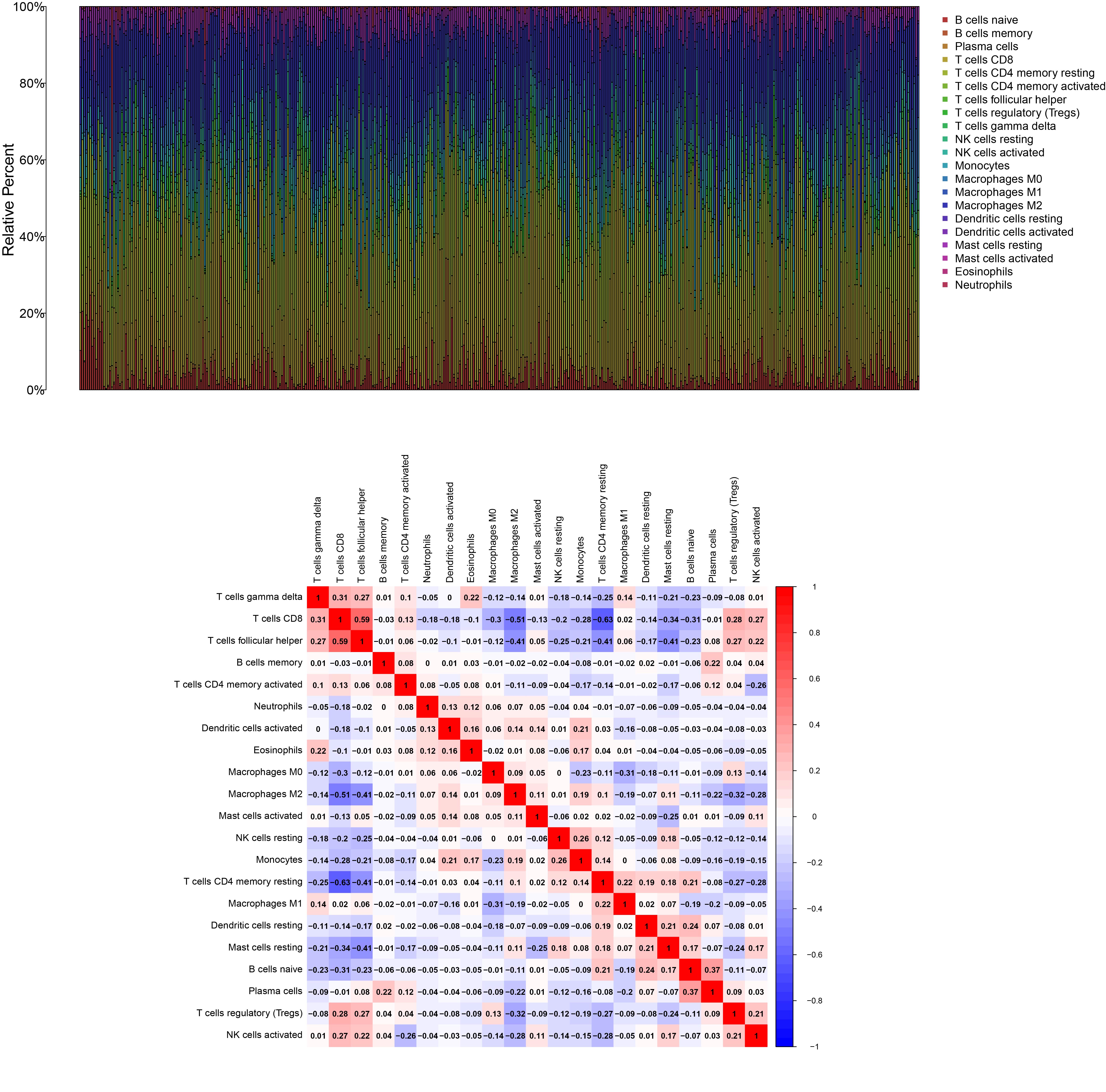

Supplement: Supplementary Figure 3 — Distribution of tumor-infiltrating immune cells (TICs) in KIRC and correlation analysis. (A) Bar plot showing the distribution of TICs in KIRC. (B) Correlation analysis of the 21 TICs in KIRC. [file Image_3.jpeg]

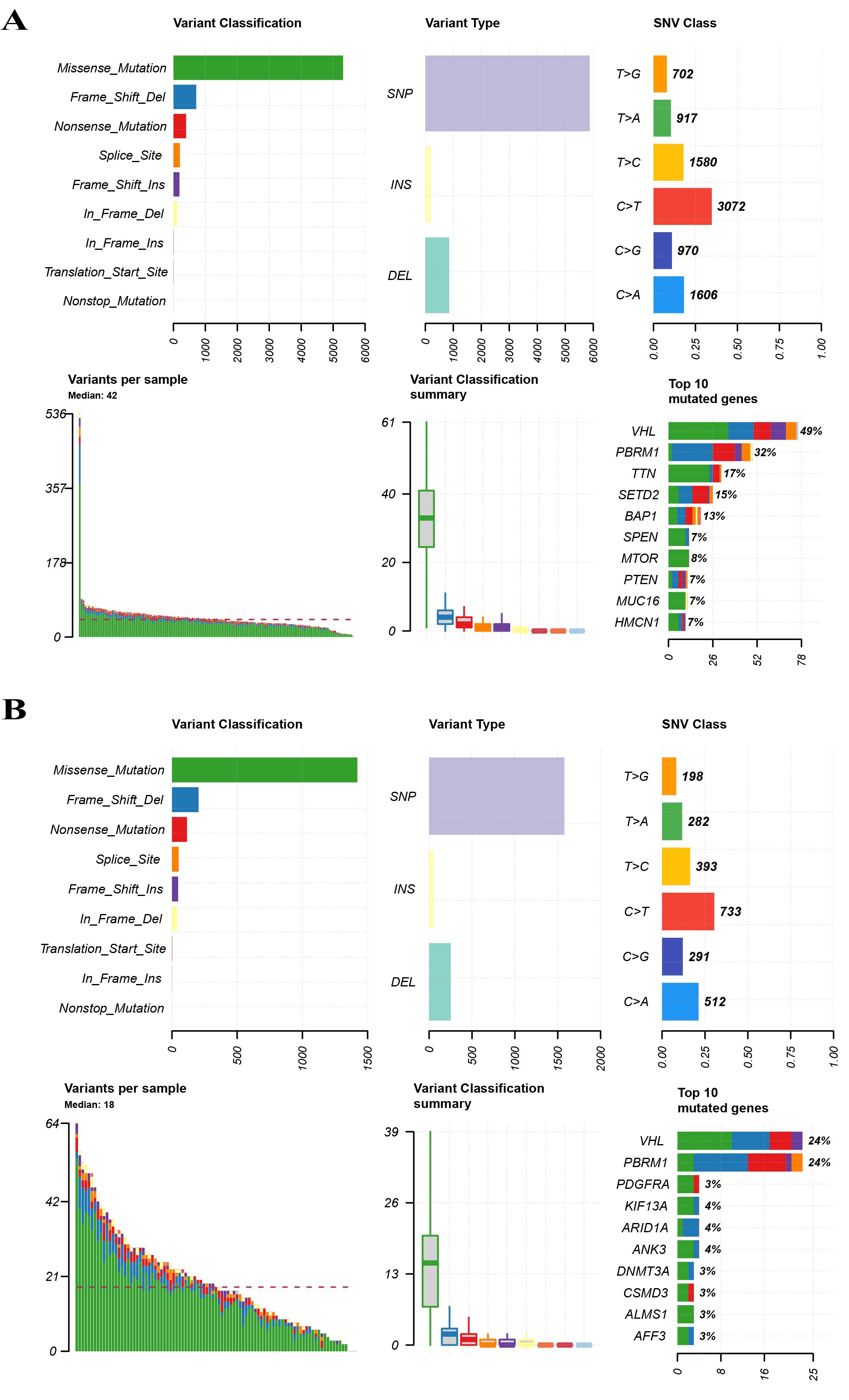

Supplement: Supplementary Figure 4 — (A) Summary of variant classification, variant type, SNV class, and variants per sample in the high-risk group. (B) Summary of variant classification, variant type, SNV class, and variants per sample in the low-risk group. [file Image_4.jpeg]
